# Exploring attachment security in a sample of Lebanese adolescents: The validation of the Arabic IPPA-R

**DOI:** 10.1371/journal.pone.0298084

**Published:** 2024-03-20

**Authors:** Pia Tohme, Nour Yaktine, Elma Nassar, Karim Badr, Ian Grey, Rudy Abi-Habib

**Affiliations:** 1 Department of Social and education Sciences, School of Arts and Sciences, Lebanese American University, Beirut, Lebanon; 2 Department of Psychology, Saint Joseph University of Beirut, Beirut, Lebanon; 3 Graduate Studies and Research Office, School of Arts and Sciences, Lebanese American University, Beirut, Lebanon; 4 SHL, Thames Ditton, Surrey, United Kingdom; 5 Department of Cognitive Sciences, United Arab Emirates University, Abu Dhabi, United Arab Emirates; St John’s University, UNITED STATES

## Abstract

Despite growing evidence supporting the importance of the quality of attachment during adolescence, no studies have been conducted to date in the Arab world due to an absence of valid and reliable tools to measure this construct in Arabic. The Inventory of Parent and Peer Attachment–Revised (IPPA-R) was devised as a self-report questionnaire in English to assess the quality of adolescent attachment to mother, father, and peers, each scale consisting of 25 items. The current study sets out to determine the psychometric properties of the Arabic IPPA-R and to explore attachment styles in Lebanon in a sample of 765 Lebanese adolescents. Results suggested a modified three-factor structure to reach satisfactory reliability of the Arabic IPPA-R, resulting in a modified questionnaire consisting of 19 items for each of mother (α = .82) and father (α = .85), and 21 items for peers (α = .89). Strict measurement invariance across gender was achieved for the IPPA-R parental forms, while only scalar invariance was achieved for the IPPA-R peers form. Overall, there were significant differences in attachment scores to mother and father, with adolescents scoring higher on attachment to mother, with both scores being significantly lower than attachment to peers. Gender differences were found on the peer scale with girls scoring significantly higher than boys. Results are interpreted from a cultural lens, emphasizing the importance of accounting for cultural, religious, and socio-economic factors in understanding adolescent attachment. This study is the first conducted in the Arab region and provides a road map to understanding gender-roles, parental expectations and adolescent perceived parenting, and their impact on adolescent attachment scores.

## Introduction

Up until the 1990s, attachment research primarily focused on the early mother-infant relationship, namely the role of maternal sensitivity on infant secure-base behavior and the future competence of secure and insecure infants. Few studies investigated this relationship into middle childhood or adolescence, partly due to a lack of valid tools measuring this construct, taking into account changes occurring during these subsequent stages of development [1]. More specifically, cognitive and emotional abilities mature, and children’s representations of caregivers become more complex, allowing for the integration of attachment representations in relation to each attachment figure into an overarching model of attachment, including peers [2–5].

Armsden and Greenberg [6] devised the IPPA, a self-report questionnaire assessing attachment to mother, father, and peers. It provides an overall attachment score for each relationship, as well as scores on three subscales: a) Trust, the degree to which the adolescent trusts the parent/peer in being available to meet their emotional needs and the extent to which they feel parents/peers trust them, b) Communication, the adolescent’s ease in discussing emotional issues and concerns with parents/peers, and c) Alienation and Anger, the perception of loneliness and not belonging to the family/peer group. A subsequent revised version (IPPA-R) was created, initially validated on a sample of participants between 16 and 20 years of age, but later observed to be valid with adolescents as young as 12 [7].

The IPPA-R has proven to differentiate between attachment relationships to various attachment figures, discriminating the role each plays in promoting positive interactions with others, psychological adjustment, and maintaining relationships across the lifespan [8]. In fact, according to a systematic review exploring measurements of attachment of middle childhood and adolescence the IPPA-R was shown to have the best psychometric properties [9]. The IPPA and IPPA-R have been translated and adapted into various languages and validated in several countries including Pakistan, China, Turkey, Peru, Cyprus, and Spain. However, findings of these studies are inconsistent, with some suggesting a one-factor structure, others suggesting either a two-factor structure, some confirming the original three-factor structure, or others suggesting a new three-factor structure.

In Pakistan, the factor structure of the IPPA-R was measured using a CFA. The authors measured a one-factor model, with an overall attachment security score; a two-factor model with trust and communication merged together into one factor, and alienation constituting the second factor; and finally, the original three-factor model with trust, communication, and alienation constituting different factors. Item loadings were comparable for the two and three-factor models, and both models had acceptable fit indices. However, given the high correlation between the three factors in the original three-factor model, the authors concluded that a two-factor model would be more suitable, in order to better be able to discriminate between the dimensions of the scale [10].

Other studies confirmed the validity of the original three-factor structure. In the Peruvian validation study of the IPPA-R, CFA results showed that the original three-factor structure had acceptable fit indices [11]. More recently, the psychometric properties of the IPPA were measured in a clinical and non-clinical sample of Cypriot adolescents [12]. Results showed that the IPPA had good psychometric properties, with Cronbach alphas ranging from good to excellent, with α = .88 for parental trust, α = .84 for parental communication, α = .82 for parental alienation, α = .90 for peer trust, α = .89 for peer communication, and α = .74 for peer alienation. Moreover, their CFA results also confirmed that the original three-factor structure would indeed be suitable for the scale [12]. In Turkey, however, a new three-factor model was suggested. Kocayörük [13] first evaluated the original three-factor model using a CFA but found poor fit indices. Thus, after running an EFA, a new structure emerged, with a shorter version of the scale. Finally, some authors have suggested a one-factor structure for the IPPA scale. In their study, Gallarin and Alonso-Arbiol [14] adapted the IPPA to Spanish and examined its factor structure. Unlike the previous studies mentioned above, the principal component analysis yielded a one-dimensional structure for the IPPA, suggesting that attachment security towards parents and peers could be measured as a whole, without having dimensions for trust, communication, and alienation. Given the inconclusive results regarding the cross-cultural validity of the original three-factor structure of the IPPA, this paper seeks to fill this gap by validating the Arabic IPPA-R in the Lebanese population.

### Attachment to parents and gender differences

Some studies have suggested that adolescents tend to be more securely attached to mothers than fathers [15–17], with the former being used as a secure base, measured in some studies by the adolescent’s perception of availability and responsiveness [17, 18]. Early research highlighted that each parent affects different aspects of the adolescent’s life [16, 19, 20]. However, studies comparing mother-adolescent and father-adolescent attachment relationships have yielded somewhat inconsistent results. On the one hand, a meta-analysis by van Ijzendoorn and Bakermans-Kranenburg [21] concluded that the distribution of adolescent attachment classifications with respect to mother and father was very similar. On the other, Doyle et al. [15] found differences in the quality of attachment to mothers and fathers, with adolescents classified as securely attached to mothers and more dismissing and fearful of fathers.

Here it is important to home in on the role of perceived caregiving behaviors in promoting coherence and integration of attachment mental representations [22]. Recent studies have found that securely attached adolescents tended to score lower on measures of perceived negative parenting and were more likely to have stronger relationships with others, considered as a source of support during emotionally loaded situations [23]. Furthermore, low perceived maternal care and parental bonding were related to difficulty with emotion expression and regulation [24], also markers of insecure attachment.

Despite the different gender-role expectations in adolescence whereby evidence suggests that girls show greater concern with interpersonal relationships and relatedness than boys who stress independence [15], gender differences in attachment have not often been addressed. Some studies reported that girls tended to be more secure and less dismissing than boys [15]; however, Ma and Huebner [25] found no gender differences in attachment to parents but highlighted that girls showed higher security scores to peers than boys. Interestingly, using self-report measures of attachment, Buist et al. [26] emphasized the importance of both, adolescent and parent genders, in elucidating these relationships whereby the quality of same-sex attachment declined during adolescence, with secure attachment to mother receiving higher scores than attachment to fathers, with the difference more marked for girls than boys.

However, one key issue that continues to attract debate concerns the role of cross-cultural variations in attachment and the potential impact of culture on attachment behaviors. Studies of diverse populations that differ from those in the original attachment studies in infancy [27–29] have shown variations in the distributions of insecure attachment rates, which may indicate positively adaptive maternal and infant behaviors in that specific cultural context [30–32]. It has been proposed that these cultural differences in attachment styles relate primarily to fundamental cultural differences in parental behaviors. These, in turn, may be influenced by additional factors, such as cultural views, affecting the respective importance of autonomy and independence of the individual, gender-role expectations and whether the society is primarily classified as individualistic or collectivistic [32]. However, less is known regarding how these same cultural differences affect attachment in adolescence.

### The role of peers and adolescent adjustment

Understanding changes in attachment in adolescence is rendered more complicated by the growing influence of peers who tend to become sources of intimacy and feedback about social behavior [33]. Laible et al. [34], using the IPPA-R, concluded that even though parents and peers may serve similar attachment functions, adolescents who scored highly on attachment measures for both relationships showed the best pattern of adjustment, echoing Howes’s [35] argument that being securely attached to more than one attachment figure is more beneficial for development than a single secure relationship. Despite the growing influence of peers, attachment scores to parents and peers have been found to be correlated, whereby the quality of the secure parent-adolescent attachment relationship provides a framework for later peer relationships based on mutuality, trust and communication [6]. In cases of insecure attachment to parents, children tend to internalize negative mental representations of themselves and other relationships [36], manifesting in adolescence as low social exploration and a reduced capacity to form new relationships [2, 8].

Furman and Buhrmester [37] concluded that the quality of the adolescents’ relationship with peers is relatively more influential on adolescent adjustment than attachment to parents. Additional research suggests that parents remain the secure base and the main attachment figure, but that peers fulfilled this role when attachment to parents tended to be less secure [38]. However, in spite of the developmental changes occurring in adolescence and the growing influence of peers, the caregiver’s availability and responsiveness to the adolescent preserves a primary importance in influencing security of parent-child attachment [39].

In terms of overall adolescent adjustment, several studies have highlighted a positive association between attachment security, and indicators of psychosocial adjustment in adolescence such as life-satisfaction, psychological adjustment and affective states [34, 40–42]. Scott and colleagues pinpointed the unique role of adolescent attachment security, albeit interrelated with other aspects of the adolescent-parent relationship, in predicting delinquent behaviors [43]. The authors suggest that these findings could reflect that a secure representation of relationships renders the adolescent more attuned to others’ feelings, needs and desires, appreciating differences in opinions, thus lessening the possibility of delinquent and problem behaviors.

### The current study

In Lebanon and the Arab world in general, research on attachment remains scarce, with no valid and reliable tools measuring adolescent attachment to parents and peers currently available. The Middle East in general has been described as being home to collectivistic cultures [44, 45], with the harmony of the society seen as a primordial goal of socialization [46–48]. Lebanon, though a Middle Eastern country, is a unique example of heterogeneity in the region as it is a country with 18 distinct communities [49].

In the Arab world, the few attachment studies have focused on adult romantic attachment, using a validated measure of attachment-avoidance and attachment–anxiety, the Experiences in Close Relationships Revised (ECR-R) [50], with high scores on either of the two scales being a marker of insecure attachment. One of the differences between the ECR-R and the IPPA-R is that the former does not provide an overall continuous score of attachment security; rather it provides scores on attachment avoidance and attachment anxiety, both markers of attachment in/security, within close relationships (romantic or parental relationship). Studies using the validated Arabic ECR-R [51] support cultural influences, with higher scores on attachment anxiety found in the Arab sample when compared to a Western sample, possibly reflecting a preoccupation with interpersonal relationships in collectivistic cultures. However, one cultural group that is notably absent from this body of research is children and adolescents.

As a first step towards examining attachment patterns and correlates of attachment in the Middle East, the current study sets out to, first, explore the psychometric properties of the Arabic version of the Revised Inventory of Parent and Peer Attachment (IPPA-R) [6] and to explore attachment scores in this sample. We will start by running a confirmatory factor analysis using the three-factor model provided by Armsden and Greenberg [6] and examine the internal consistencies of the IPPA-R scores. Second, we will assess the convergent validity of the IPPA-R and expect to find significant negative correlations with the attachment-avoidance and attachment-anxiety subscales of the ECR-R, reflecting insecure attachment. Third, we will examine construct validity and expect that total scores on the IPPA-R will be negatively correlated to perceived negative parenting and a measure of social maladjustment, namely social difficulties scores. The second aim of this study is to explore attachment patterns in this sample, and we hypothesize that 1) adolescents’ scores to both parents and peers, based on the total IPPA-R overall continuous attachment score, will be significantly correlated, but we do not expect significant differences in attachment scores, and 2) Finally, we set out to explore gender differences in adolescent attachment.

## Method

### Participants

The sample initially consisted of 790 school students. After deletion of age outliers, 765 participants remained, 37.4% of which were boys (*N* = 286) and 46.9% of which were girls (*N* = 359), with 15.7% of participants not answering the gender question. Adolescents were aged between 12 and 18 years (*M* = 15.00, *SD* = 1.97), from grades 6 to 12 (32.3% in elementary grades and 58.7% in secondary grades, with 9% of participants not answering this question). Fifty-three percent of participants were from public schools while the rest were from private schools, from both the capital Beirut and other areas in Lebanon. Parental status was rated based on adolescents’ answers on a categorical yes/no question as to whether or not they lived with both parents; 76% reported living with both. The only inclusion criteria besides age was being a fluent Arabic speaker.

### Procedure

After receiving the validation approval from the IPPA authors, two certified translators proceeded to the Arabic translation and back translation. Two certified clinical psychologists compared the English versions, leading to some minor changes in the choice of Arabic words used.

After receiving approval from the Ethics Institutional Review Board, data collection began within elementary and secondary classrooms from 11 schools, 4 private and 7 public. First, the researchers explained the study objectives to the school principals who signed the consent upon agreement. Parents read the information sheet and gave approval regarding their child’s participation. Students whose parents gave consent signed an assent form before filling the booklet. Questionnaires were distributed during class time, requiring approximately 25 minutes to fill out. Researchers were present during data collection to answer participants’ questions.

### Measures

The *Inventory of Parent and Peer Attachment-Revised* (IPPA-R) [6] is a 5-point Likert scale self-report questionnaire initially devised to assess the quality of adolescents’ attachment relationships to parents and peers. The revised version contains 25 items for each of mother, father, and peers, thus yielding 3 continuous attachment scores. Items can also be sorted in 3 main subscales: Degree of Mutual *Trust* (10 items, “My mother respects my feeling”), Quality of *Communication* (9 items, “I like to get my mother’s point of view on things I’m concerned about”) and Extent of *Anger and Alienation* (6 items, “I get upset a lot more than my mother knows about”). Greenberg and Armsden [52] suggest using the revised version when possible, with Cronbach’s Alpha of *α* = .87 for attachment to mother, *α* = .89 for father and *α* = .92 for peers. Test-retest reliabilities were .93 for parent attachment and .86 for peers in a sample of young adults. Concurrent validity was established as higher attachment scores were related to less conflict with parents and less adolescent loneliness [53]. Discriminant validity was established as IPPA scores distinguished delinquents from non-delinquents among 12- to 17-year-olds [54].

The *Experiences in Close Relationships-Revised* (ECR-R) [50] assesses self-reported attachment anxiety and avoidance in emotionally intimate relationships, including 36 items each rated on a 7-point scale. Each subscale is scored by computing the average of answers of 18 items. Initially this scale was devised enquire about romantic relationships; however, Fraley and colleagues explain that it can be used to measure avoidance and anxiety within parental relationships as well (http://labs.psychology.illinois.edu/~rcfraley/measures/ecrr.htm). Therefore, for this study, sample items include “I don’t like telling my parents how I feel deep down inside” for attachment avoidance and “I’m worried that my parents might want to leave me” for attachment anxiety. The Arabic ECR-R has been validated in the Lebanese context. Hijazi [55] reported high internal consistencies for the anxious and avoidant dimensions of the Arabic ECR-R (*α* = .84 and *α* = .86, respectively), and an inter-correlation of *r* = .26, *p* < .01 with the Arabic CES-D. The two subscales were found to correlate minimally at .03 in Kazarian [56], at .05 in Kazarian and Martin [57], and to correlate higher at .42 in Sibley and Liu [58]. Given that participants were required to answer IPPA attachment questions separately for mother, father and peer, questions of the ECR-R in this study were asked in a generalized manner about parents in order to answer the questions once. In this sample, internal consistencies were of *α* = .82 for the attachment-anxiety scale and *α* = .61 for the attachment-avoidance scale, and the two subscales were found to be significantly correlated at .48.

The *Measure of Parental Style* (MOPS) [59] was used to assess perceived negative parenting. There are 15 statements in total (e.g. “is overprotective of me”, “ignored me”), scored once about each caregiver on a scale from 1 to 4, the sum of which provides two total scores reflecting the level of negative parenting experienced by adolescents, one for each parent. Lower scores reflect lower perceived negative parenting. Internal reliabilities for this sample were high with *α* = .90 for mother and *α* = .91 for father.

The *Strengths and Difficulties Questionnaire* (SDQ–Child version) [60] is a brief behavioral screening questionnaire for 11- to 18-year-olds. The 25-item questionnaire assesses five behavioral traits, four of these relating to problem behaviors (Conduct Problems, Emotional Problems, Hyperactivity-Inattention and Peer Problems), each consisting of the sum of scores of 5 items, and one relating to a strength behavior (Prosocial Behavior). The problem behaviors subscale, Total Difficulties, includes statements such as “I am easily distracted” and “I am usually on my own”. The SDQ is extensively supported, with a good internal consistency of .73 and test-retest reliability of .62 [61]. The validated Arabic version of the SDQ is provided on the author’s website to be freely used. For this study, we only used the Total Difficulties scale, with an internal consistency of *α* = .81.

### Statistical analysis

First, the distribution of key variables was inspected visually using the qq plots and statistically using the Kolmogorov-Smirnov test. Since normality was not achieved, a confirmatory factor analysis based on the maximum likelihood estimation with the Satorra-Bentler correction and robust standard errors was conducted to test the three-factor theoretical structure of the IPPA-R, which we call Model Zero (M0). Several robust fit indices were used to assess CFA model fit: the root-mean-squared error of approximation (RMSEA), the standardized root mean square residual (SRMR), the comparative fit index (CFI), Tucker-Lewis index (TLI), and the chi-square test statistic. Hu and Bentler [62] established cutoff criteria as follows: CFI and TLI should be greater than 0.90, SRMR less than 0.08 and a RMSEA below 0.05, noting that Steiger [63] relaxed this threshold to 0.07. Next, the resulting factor loadings of M0 were inspected. A modified model (M1) was created by removing items with a factor loading below .400. Models were compared by the chi-square difference test, whereby a model is judged better than the other if the former significantly reduces the chi-square statistic.

Convergent and construct validity were established by examining correlations between the IPPA-R scores and other relevant measures. In addition, Spearman intercorrelations between IPPA-R scores were investigated, p-values were adjusted for multiple comparisons using the Bonferroni correction.

Confirmatory factor analysis for measurement invariance across gender was investigated over four hierarchical levels: the first is configural invariance testing the suitability of the global structure across groups and is considered as the baseline model; the second is metric invariance and tests whether factor loadings are equal across groups; the third is scalar invariance testing equality in factor loadings and intercepts across groups; and the fourth and highest level of invariance is strict invariance and tests whether the measurement at the item level is identical. Strict invariance being too strict to achieve in practice, scalar invariance remains the commonly accepted pre-requisite for mean comparisons. Evaluating invariance from one level to another is done by comparing each model to the previous one and was assessed using the recommendations by Chen [64]. The criteria for testing invariance are having a difference in CFI > -0.01, a difference in RMSEA < 0.015, and a difference in SRMR less than 0.03 for loading invariance and less than 0.01 for intercept or residual invariance. When at least scalar invariance was achieved, independent samples t-tests were conducted to study gender differences in the IPPA-R scores. All statistical analyses were done using the software R version 4.0.3.

## Results

### Confirmatory factor analyses

**Fig 1:**
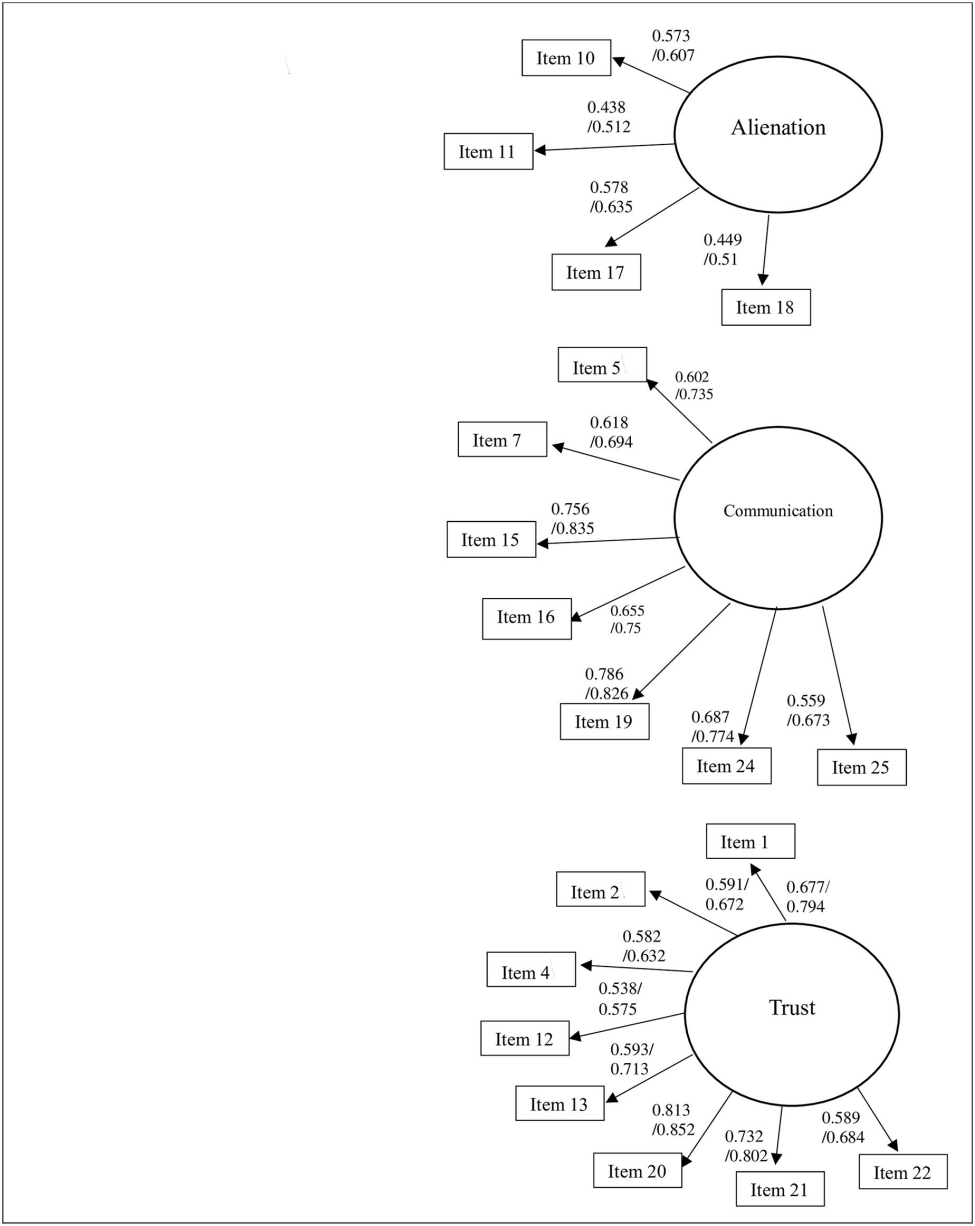
Confirmatory factor analysis for mother and father.

This study set to investigate the psychometric properties of the Arabic version of the IPPA-R. The confirmatory factor analysis showed that the theoretical three-factor model of the IPPA-R Mother and IPPA-R Father had satisfactory fit indices, nevertheless some items had very low or negative loadings on their corresponding theoretical factor (Fig 1). These items, specifically having loadings less than .400, were excluded, resulting in a modified model called M1. The items that were removed from both parental forms are: item 3 “I wish I had a different parent”, item 6 “I feel it’s no use letting my feelings show around my parent”, item 8 “Talking over my problems with my parent makes me feel ashamed or foolish”, item 9 “My parent expects too much from me”, item 14 “My parent has his own problems, so I don’t bother him with mine”, and item 23 “My parent doesn’t understand what I’m going through these days”. Additional reliability analyses showed that deleting these items increased the global Cronbach’s alpha of the maternal and parental forms from .70 and .66 respectively to .82 and .85. The resulting parental scales contained 19 items, with Cronbach’s alpha ranging between 0.57 and 0.90 (Table 1).

**Table 1:** Cronbach’s alpha levels.

	Cronbach’s alpha	Number of items
IPPA-R Mother	0.82	19
IPPA-R Mother Communication	0.85	7
IPPA-R Mother Trust	0.85	8
IPPA-R Mother Alienation	0.57	4
IPPA-R Father	0.85	19
IPPA-R Father Communication	0.90	7
IPPA-R Father Trust	0.90	8
IPPA-R Father Alienation	0.65	4
IPPA-R Peers	0.89	21
IPPA-R Peers Communication	0.87	8
IPPA-R Peers Trust	0.90	10
IPPA-R Peers Alienation	0.62	3

**Fig 2:**
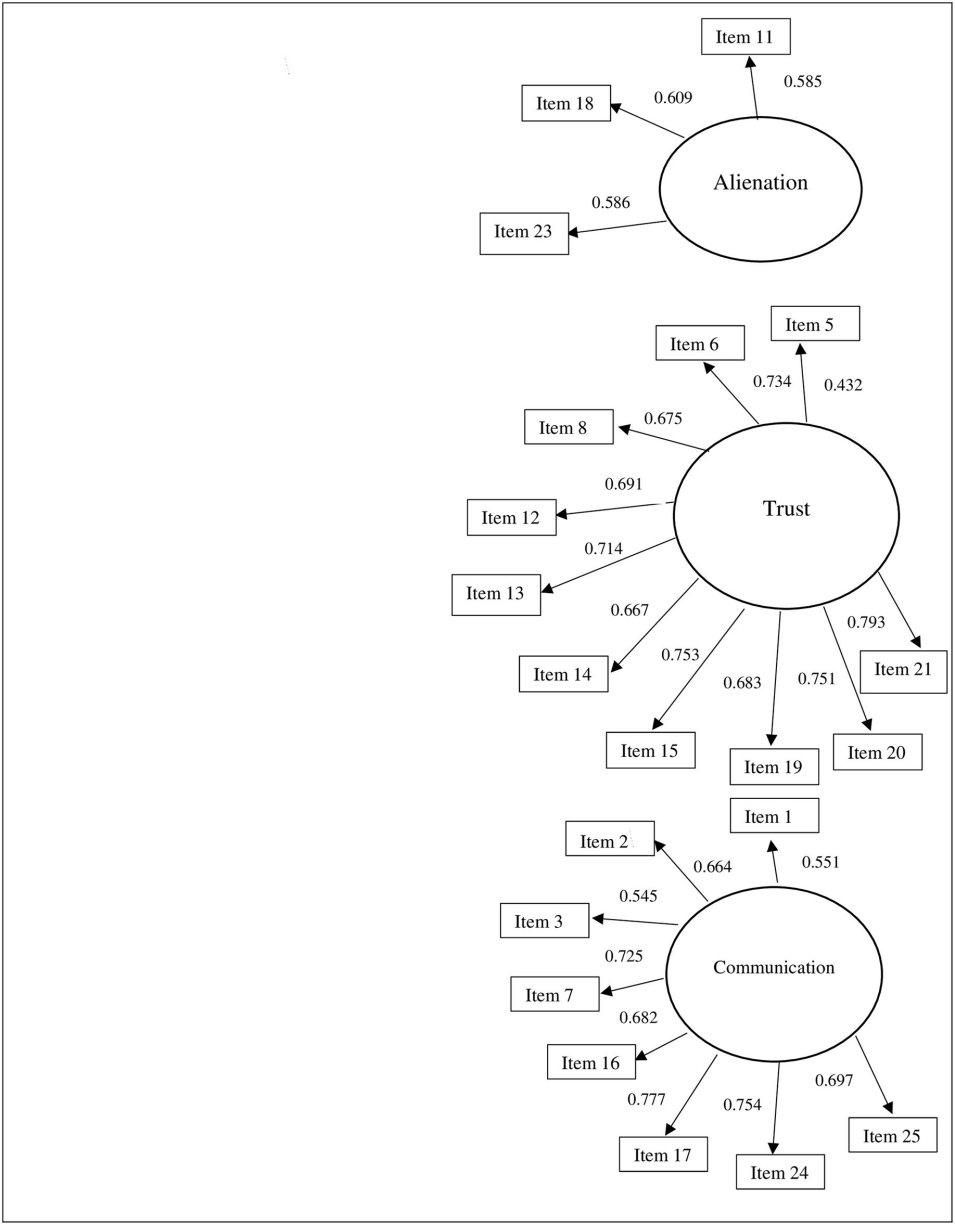
Confirmatory factor analysis for peers.

Concerning the IPPA-R Peers, the first CFA (Fig 2) showed satisfactory fit for the three-factor model with a very good overall Cronbach’s alpha of .84. Further investigation of the loadings suggested a modified model M1 without the following items: item 4 “Talking over my problems with my friends makes me feel ashamed or foolish”, item 9 “I feel the need to be in touch with my friends more often”, item 10 “My friends don’t understand what I’m going through these days”, and item 22 “I get upset a lot more than any friends know about”. As a result, Cronbach’s alpha increased to 0.89. The resulting peer scale contained 21 items (Table 1).

Models M0 and M1 were compared via the chi-square difference test. Table 2 shows that the modified model M1 outperformed the theoretical model M0 for the parental and peer forms.

**Table 2:** Confirmatory factor analyses for the theoretical three-factor structure (M0) and the modified one (M1) for each of the three IPPA-R forms.

	Model	RMSEA (95% CI)	CFI	TLI	SRMR	χ2 (df)	Model Comparison	Δ χ2 (df)
IPPA- Mother	M0	.046 (.042,.051)	0.919	0.911	0.049	616.93 (272)		
	M1	.044 (.038,.052)	0.954	0.947	0.040	315.54 (149)	M0—M1	304.88 (123)
IPPA-Father	M0	.060 (.056,.065)	0.909	0.900	0.054	858.70 (272)		
	M1	.060 (.054,.067)	0.945	0.937	0.041	452.67 (149)	M0—M1	407.39 (123)
IPPA-Peers	M0	.055 (.050,.060)	0.920	0.912	0.056	731.34 (272)		
	M1	.059 (.053,.065)	0.935	0.926	0.047	522.73 (186)	M0—M1	203.70 (86)

Note. All the conducted chi-square difference tests showed to be significant.

### Convergent and construct validity

To explore the convergent validity of the Arabic IPPA-R, we ran Spearman correlations between each of the IPPA-R (total scores and the three subscales) and a similar measure of the attachment construct, the Experiences in Close Relationships-Revised (ECR-R). Significant correlations were found between the two attachment measures (Table 3).

**Table 3:** Correlations between IPPA-R and its subscales, ECR-R, MOPS, and SDQ scores.

	ECR-R Anxiety	ECR-R Avoidance	MOPS Mother	MOPS Father	Total Difficulties
IPPA-R Mother	-0.22	-0.58	-0.32	-0.25	0.03
IPPA-R Mother Communication	-0.25	-0.63	-0.35	-0.29	0.02
IPPA-R Mother Trust	-0.32	-0.60	-0.41	-0.33	0.00
IPPA-R Mother Alienation	0.33	0.44	0.40	0.34	0.04
IPPA-R Father	-0.23	-0.51	-0.20	-0.33	-0.03
IPPA-R Father Communication	-0.26	-0.54	-0.24	-0.35	-0.03
IPPA-R Father Trust	-0.32	-0.52	-0.31	-0.47	-0.06
IPPA-R Father Alienation	0.38	0.42	0.38	0.49	0.06
IPPA-R Peers	-0.16	-0.24	-0.19	-0.18	0.00
IPPA-R Peers Communication	-0.15	-0.21	-0.19	-0.16	0.03
IPPA-R Peers Trust	-0.25	-0.31	-0.25	-0.23	-0.07
IPPA-R Peers Alienation	0.32	0.29	0.27	0.22	0.09

Construct validity was investigated, looking for correlations between IPPA-R scores (and its subscales) and MOPS and SDQ Difficulties scores (Table 3). Significant correlations were found between IPPA-R Mother and MOPS Mother, *r*(586) = -.32, *p* < .001 and between IPPA-R Father and MOPS Father, *r*(586) = -.25, *p* < .001. No significant correlations were found between the IPPA-R and the SDQ.

### Measurement invariance and gender comparisons

Measurement invariance was conducted using confirmatory factor analysis to test similarity of the IPPA-R structure across gender. Four models were constructed and compared. First, the configural model with the same suggested structure M1 for each gender, the second is the metric model constraining loadings on factors across gender, the third is the scalar model constraining loadings and intercepts across gender, the fourth and last is the strict model constraining loadings, intercepts, and residuals across gender. Comparison of models showed a change in CFI above -0.01, as well as changes in RMSEA below 0.015 and in SRMR below 0.03 for loading invariance and below 0.01 for intercept and residual invariance, except in the strict vs scalar comparison in the case of the IPPA-R peers. As a conclusion, the highest form of measurement invariance, which is strict invariance, was established for the parental forms, while only scalar invariance was achieved in the case of the IPPA-R peers. Partial strictness was investigated to adjust for non-invariant items and was not achieved even after freeing more than 50% of item estimates. Results of model comparisons can be found in Table 4.

**Table 4:** Measurement invariance of the M1 structure across gender for each of the three forms of the IPPA-R.

	Model	RMSEA	95% CI	CFI	SRMR	Model comparison	ΔRMSEA	ΔCFI	ΔSRMR
IPPA-R Mother	Configural	0.052	0.044,0.060	0.942	0.047				
	Metric	0.053	0.045,0.061	0.936	0.060	metric—config	0.001	-0.006	0.013
	Scalar	0.056	0.048,0.063	0.926	0.062	scalar—metric	0.003	-0.010	0.002
	Strict	0.054	0.046,0.062	0.926	0.064	strict—scalar	-0.002	0.000	0.002
IPPA-R Father	Configural	0.063	0.050,0.070	0.942	0.046				
	Metric	0.062	0.055,0.070	0.940	0.056	metric—config	-0.001	-0.002	0.010
	Scalar	0.064	0.057,0.071	0.934	0.058	scalar—metric	0.002	-0.006	0.002
	Strict	0.063	0.056,0.070	0.931	0.059	strict—scalar	-0.001	-0.003	0.001
IPPA-R Peers	Configural	0.062	0.055,0.070	0.927	0.052				
	Metric	0.063	0.056,0.070	0.923	0.065	metric—config	0.001	-0.004	0.013
	Scalar	0.061	0.054,0.068	0.923	0.065	scalar—metric	-0.002	0.000	0.000
	Strict	0.071	0.064,0.077	0.892	0.069	strict—scalar	0.010	-0.031	0.004

Given that scalar invariance was achieved for the three forms of the IPPA-R, we conducted independent samples t-test to study gender differences in adolescent attachment. Boys and girls did not score significantly differently on attachment to mother, *t*(610.6) = 0.40, *p* = 0.69, nor to father, *t*(612.9) = 0.26, *p* = 0.79. However, girls (M = 78.42, SD = 12.68) scored significantly higher than boys (M = 71.72, SD = 12.77) on overall IPPA-R Peer, with *t*(579.8) = 6.27, d = 0.50. Boys (M = 3.05, SD = 1.13) scored significantly higher than girls (M = 2.76, SD = 1.27) on ECR-R Avoidance, with *t*(6245) = 3.05, d = 0.24. No significant gender differences were obtained on the ECR-R Anxiety factor (p = 0.15) (Table 5).

**Table 5:** Descriptive statistics and independent samples t-tests across gender.

	Boys	Girls	T-test	p
	M ± SD	M ± SD		
IPPA				
Attachment-mother	67.32 (± 10.58)	67.65 (±10.56)	0.40	0.69
Communication-Mother	26.59 (± 6.03)	26.86 (6.44)	0.55	0.59
Trust-mother	33.08 (6.03)	33.30 (5.85)	0.48	0.63
Alienation-mother	7.65 (3.08)	7.50 (2.95)	0.66	0.51
Attachment-father	62.93 (12.77)	62.66 (12.86)	0.26	0.79
Communication-father	24.10 (7.38)	23.11 (7.65)	1.67	0.10
Trust-father	30.72 (7.52)	31.60 (7.55)	1.48	0.14
Alienation-father	8.11 (3.60)	7.95 (3.40)	0.58	0.56
Attachment-peers	71.72 (4.07)	78.42 (12.68)	6.27	< 0.001
Communication-peers	27.72 (7.05)	31.40 (6.68)	6.76	< 0.001
Trust-peers	37.90 (8.55)	41.37 (7.57)	5.39	< 0.001
Alienation-peers	6.11 (2.68)	5.65 (2.61)	2.22	0.03
ECR				
Attachment anxiety	3.33 (0.95)	3.22 (1.08)	1.46	0.15
Attachment avoidance	3.05 (±1.13)	2.76 (±1.27)	3.05	0.002

It was hypothesized that adolescents’ scores to both parents and peers will be significantly correlated but that there would not be any significant differences in scores. IPPA-R Mother total scores and IPPA-R Father total scores were found to be positively correlated, with *r* = .58, *p* < .001, and IPPA-R Peers total scores were significantly correlated with IPPA-R Mother scores, with *r* = .25 and IPPA-R Father scores with *r* = .26, *p* < .001 for both. Descriptive statistics of the Arabic IPPA-R revealed that, overall, adolescents scored highest on the Arabic IPPA-R Peer, followed by IPPA-R Mother, with the lowest scores on IPPA-R Father. Differences were significant between IPPA-R Peer (*M* = 75.73, *SD* = 13.62) and Father (*M* = 63.42, *SD* = 13.14), with *t* (688) = 19.36, *d* = 0.74, between IPPA-R Peer and IPPA-R Mother (*M* = 67.55, *SD* = 10.65), *t*(737) = 14.34, *d* = 0.5, and between IPPA-R Mother and Father, with *t*(672) = 9.64, *d* = 0.37.

## Discussion

### Factor analysis of the Arabic IPPA-R

This study was the first to explore adolescent attachment in the Arab world in a sample of 765 Lebanese adolescents. The main aim was to investigate the psychometric properties of the Arabic version of the IPPA-R, a self-report questionnaire yielding a continuous score of adolescent attachment to mother, father, and peers. CFA results suggest that the three-factor structure of the scale is suitable, with some modifications.

Indeed, some items did not load on the model of attachment to parents and had to be deleted such as question 9, “My mother/father expects too much from me”. This can be understood through a cultural lens, as parents from collectivistic cultures have been found to endorse the authoritarian parenting style, which was found to be positively correlated to group cohesion [47]. Indeed, Hatab and Makki [65] found that Lebanese adolescents tend to follow their parents’ direction in terms of their perceptions of others, values, and beliefs, reflecting a pattern of mutual interaction which tends to be stable across the lifespan. The deletion of items resulted in a revised Arabic IPPA-R containing 19 items for each of the mother (*α* = .82) and father (*α* = .85) scales.

Factor analysis of the attachment to peers scale revealed that high Cronbach Alpha (*α* = .84) if all 25 questions remained; however, further investigation of the loading led to the modification of the subscale and the removal of 4 items, leading to a higher Cronbach Alpha (*α* = .89). One such item includes question 22, “I get upset a lot more than any friends know about.” It can be argued that this item does not significantly contribute to the overall attachment style as previous research has shown that Lebanese young adults tended to be more avoidant and anxious when compared to their Western counterparts [51]. In other words, it can be posited that, from a cultural perspective, individuals are encouraged to downplay some emotions in favor of being preoccupied with the group’s needs. This may be associated with a reduced emotional expression, irrespective of the quality of attachment.

### Convergent validity of the Arabic IPPA-R

Investigating the convergent validity of the Arabic IPPA-R, negative significant correlations were found between IPPA-R total scores to both parents and ECR-R scores on attachment anxiety and avoidance, suggesting that securely attached adolescents were less likely to be anxious and avoidant within their relationships. Similarly, the ECR-R subscales were found to be negatively correlated with the IPPA-R Trust-Communication parental subscale and positively correlated with the IPPA-R Alienation subscale. This is in line with theories conceptualizing secure attachment as manifested by a valuing of attachment relationships, feeling accepted, an acknowledgement of the impact of separation, a need for comfort and support from attachment figures in times of distress, and an ability to discuss difficult and emotionally-loaded events without being overwhelmed by feelings [3, 4, 66].

Correlations between the two questionnaires ranged from low to moderate, in keeping with the idea that, despite both measures assessing attachment, the IPPA-R and the ECR-R tap into different aspects of that construct, with the former looking at the explicit evaluation of the quality of different attachment relationships [6, 52] and the latter focusing on self-reported attachment anxiety and attachment avoidance in relation to the general experience of emotionally intimate relationships [50].

Interestingly and contrary to our expectations, the overall IPPA-R score to peers was not significantly correlated with attachment anxiety. These findings could evoke that peers exert an external influence on adolescents, suggesting that different pathways characterize attachment to parents and attachment to peers. Given that Lebanon has been characterized as a collectivistic culture [67], it can be posited that having close relationships with peers is assumed and self-evident in this type of culture, therefore, it does not necessarily relate to an anxiety emerging in the context of the parent-adolescent relationship.

### Construct validity: Attachment, parenting, and adolescent adjustment

We examined construct validity by exploring the relationship between attachment and another measure of parenting, perceived negative parenting. As expected, findings suggested that the lower adolescents scored on perceived negative parenting, the more likely they were to be secure in their attachment style. This echoes findings associating sensitive parenting with secure attachment in childhood, as sensitive mothers are more likely to be responsive to their children’s signals and needs [21]. Dix [68] emphasized the necessity for affective communication from parents seen as promoting more understanding on the part of the adolescent in the processing of messages and intentions, giving them the opportunity to evaluate different points of view. Granic, Dishion and Hollenstein [69] extended this idea by arguing that parents need to be more flexible and allow adolescents to make their own decisions, trusting them to make the right choices, in order to, with time, and through an appropriate feedback process, give them more confidence and competence.

Surprisingly, no significant correlation was found between the SDQ Total Difficulties scale and the IPPA-R scores, contradicting the literature associating these two measures [70–73]. This could suggest that adolescents’ reported social and emotional difficulties in the Lebanese setting could relate to other factors such as mental health problems [74, 75], family instability [76, 77], or the socioeconomic context [78].

Noteworthy is the significant correlation, despite being small, between the SDQ Total Difficulties scale and IPPA-R Father Alienation, as well as IPPA-R Peers Alienation. This could suggest that social difficulties and problem behaviors increase the more alienation and isolation the adolescent perceives from peers and their father. This taps into the different roles played by the various attachment figures during adolescence in predicting social adjustment. However, due to the low correlations, these results should be replicated in future studies.

### Adolescent attachment and gender differences

As this study was the first to look at adolescent attachment in Lebanon, its secondary aim was to explore attachment scores in this sample. In line with our expectation, a moderate significant correlation between attachment to mother and attachment to father was found. Investigating differences between attachment to mothers and fathers, contrary to our expectations, we found that scores were significantly different between each of the caregivers, with adolescents scoring highest on the IPPA-R Peers, followed by IPPA-R Mother and the lowest on IPPA-R Father. Our findings revealed high effect sizes for differences between IPPA-R Peers and scores on the parental IPPA-R suggesting the growing influence of peers as sources of intimacy during this stage [33]. Blos [79] argued that the separation from parents can lead to a sensation of aloneness, rendering the adolescent more likely to turn to peers as a source of belonging and comfort, providing support as the adolescent identifies and spends time with people who have shared ideas and goals, enabling him/her to try new roles and identities within the group [80]. More recent findings converge with this theory suggesting that peers are perceived as a source of emotional support and proximity seeking during adolescence, emphasizing the latter construct as conceptualized differently than in attachment measures in infancy [8, 38, 81]. The differences between IPPA-R Mother and IPPA-R Father scores revealed medium effect sizes, tapping in the different perceived roles of each of the parents, with mothers seen as providers of emotional support, essential during this developmental stage [15]. It is interesting to further explore this finding to delve deeper into the clinical impact and significance of higher attachment to peers, qualitatively comparing adolescents’ responses regarding their perception of their attachment to parents and peers.

Exploring gender differences in attachment scores, no significant differences were found in scores towards parents. However, girls scored significantly higher than boys on attachment to peers solely, in line with previous findings by Ma and Huebner [25]. These results could be interpreted in terms of gender-role expectations developing during adolescence. In fact, girls in our sample were found to be significantly less avoidant than boys, suggesting that men are less comfortable being close to others and engage in emotional discussions. This is in line with cultural factors, whereby men in collectivistic patriarchal cultures are taught to dismiss their feelings whereas women are encouraged to express them [55], echoing previous findings in the Arab region depicting adolescent girls as scoring higher than boys on interpersonal intelligence [82], or making more use of emotion regulation strategies, through seeking social support, as a coping style in stressful events [83, 84]. However, it would be interesting for future studies to explore other factors explaining these gender differences, as gender-role expectations have been found to vary based on many factors including patriarchal societies, parents’ family roles, and religion among others [85]. Furthermore, future studies could focus on accounting for the interaction between parent and adolescent gender in further elucidating these findings.

### Limitations

Despite the uniqueness of this study, findings should be interpreted in light of some limitations. First, the type of measures used included self-report questionnaires, criticized for providing limited closed-ended options, easily swayed by mood, and solely tackling conscious representations. Furthermore, the MOPS was not previously validated in Arabic, despite high internal consistencies in our sample. Also, the SDQ scale was positioned at the end of the booklet of questionnaires, thus increasing the number of missing data. It would therefore be of interest to replicate these findings. Finally, it would be interesting to conduct further studies replicating our findings with the updated subscales of the Arabic IPPA-R, as well as exploring similarities and differences during the various stages of early, mid and late-adolescence as age has been found to affect attachment security and attachment avoidance [51]. In addition, some scholars have classified Lebanon as both an individualistic and collectivistic culture [67]; it would therefore be intriguing to examine cultural orientation, especially in light of globalization and westernization, as well as its impact on Lebanese adolescents’ attachment representations. Finally, it would be of interest to explore attachment using interview narratives, delving into the effects of gender roles on emotional expression and attachment coherence, as well as adolescent perceptions and expectations of different attachment figures.

## Conclusion

In summary, this study was the first to be conducted in the Arab region exploring attachment security in adolescents, and culturally adapting and validating the Arabic IPPA-R in the Lebanese population. Our results suggest that the Arabic IPPA-R shows good psychometric properties and is a valid tool to be used on Arabic speaking adolescents. Our findings also emphasize the importance of culture in the conceptualization of attachment, namely the operationalization of communication style, trust, and alienation in the context of parental and peer relationships. This study thus provides an initial map of Arab adolescent attachment representations and offers initial directions towards future work delving more in depth into the various cultural influences on the adolescent’s construction of internal working models of the mind.
